# Bidirectional ventricular tachycardia and cardiogenic shock caused by acute haemorrhagic necrosis of pheochromocytoma: a case report

**DOI:** 10.1093/ehjcr/ytaf664

**Published:** 2025-12-22

**Authors:** Alejandro Manuel López-Pena, Charigan Abou Jokh-Casas, Jorge Armesto-Rivas, Gonzalo De Urbano-Seara, Carlos González-Juanatey

**Affiliations:** Cardiology Department, Hospital Universitario Lucus Augusti, 27003 Lugo, Spain; CardioHULA Research Group, Fundación Instituto de Investigación Sanitaria de Santiago de Compostela FIDIS, 27003 Lugo, Spain; Cardiology Department, Hospital Universitario Lucus Augusti, 27003 Lugo, Spain; CardioHULA Research Group, Fundación Instituto de Investigación Sanitaria de Santiago de Compostela FIDIS, 27003 Lugo, Spain; Cardiology Department, Hospital Universitario Lucus Augusti, 27003 Lugo, Spain; CardioHULA Research Group, Fundación Instituto de Investigación Sanitaria de Santiago de Compostela FIDIS, 27003 Lugo, Spain; Cardiology Department, Hospital Universitario Lucus Augusti, 27003 Lugo, Spain; CardioHULA Research Group, Fundación Instituto de Investigación Sanitaria de Santiago de Compostela FIDIS, 27003 Lugo, Spain; Cardiology Department, Hospital Universitario Lucus Augusti, 27003 Lugo, Spain; CardioHULA Research Group, Fundación Instituto de Investigación Sanitaria de Santiago de Compostela FIDIS, 27003 Lugo, Spain

**Keywords:** Pheochromocytoma, Bidirectional ventricular tachycardia, Catecholamine, Differential diagnosis, Cardiogenic shock, Case report

## Abstract

**Background:**

Pheochromocytoma is associated with serious cardiovascular complications resulting from the effect of catecholamines, such as ventricular arrhythmias, cardiomyopathy with left ventricular dysfunction, and cardiogenic shock. Bidirectional ventricular tachycardia (BVT) is a rare but potentially fatal form of polymorphic tachycardia associated with elevated sympathetic tone.

**Case summary:**

A 27-year-old Caucasian woman was admitted to the intensive care unit with cardiogenic shock. The electrocardiogram showed BVT, and the transthoracic echocardiogram showed a dilated left ventricle with severe systolic dysfunction. Computed tomography revealed an adrenal mass with haemorrhage, which was confirmed by abdominal magnetic resonance imaging. The patient underwent a laparoscopic right adrenalectomy after pre-operative preparation with alpha–beta blockade, with subsequent histopathological confirmation of the diagnosis.

**Discussion:**

The acute presentation of haemorrhagic necrosis of a pheochromocytoma is a rare and potentially lethal condition. Its diagnosis can be challenging, especially in cases with atypical manifestations. However, the presence of BVT might serve as a crucial guide for its identification. Early recognition of this condition is essential, as it significantly impacts the patient's prognosis.

Learning points

- Given its narrow differential diagnosis, bidirectional ventricular tachycardia should prompt a thorough investigation into underlying causes such as pheochromocytoma, especially in atypical or complex clinical scenarios.
- Given the poor prognosis associated with cardiogenic shock, early recognition of pheochromocytoma and timely, aggressive, and appropriate medical treatment are essential to improve clinical outcomes.

## Introduction

Pheochromocytoma is a rare neuroendocrine tumour that originates in the chromaffin cells of the adrenal medulla or paraganglia.^1,2^ Its clinical presentation is variable, characterized mainly by typical symptoms such as headache, sweating, palpitations, and high blood pressure.^1^ In a few cases, initial manifestations include severe and potentially life-threatening cardiovascular complications attributed to the effects of secreted catecholamines, such as ventricular arrhythmias, heart failure with left ventricular dysfunction, or cardiogenic shock.^3^

Bidirectional ventricular tachycardia (BVT) is a rare but potentially lethal form of polymorphic ventricular tachycardia (PVT). It is associated with elevated sympathetic tone and is characterized by beat-to-beat alternation of the QRS complex axis. Its predominant causes include cardiac glycoside toxicity and catecholaminergic polymorphic ventricular tachycardia (CPVT). It has also been documented in other conditions related to intracellular calcium overload, which predispose cardiac myocytes to late repolarization and triggered activity.^4^ Although pheochromocytoma usually causes hypertension, 20% of patients may experience hypotension, and 2% may develop cardiogenic shock.^5^

This case describes an unusual presentation of pheochromocytoma in the form of BVT. Given the poor prognosis of cardiogenic shock, early recognition of this entity and timely management are essential to improving clinical outcomes.^6,7^

## Summary figure

**Figure:**
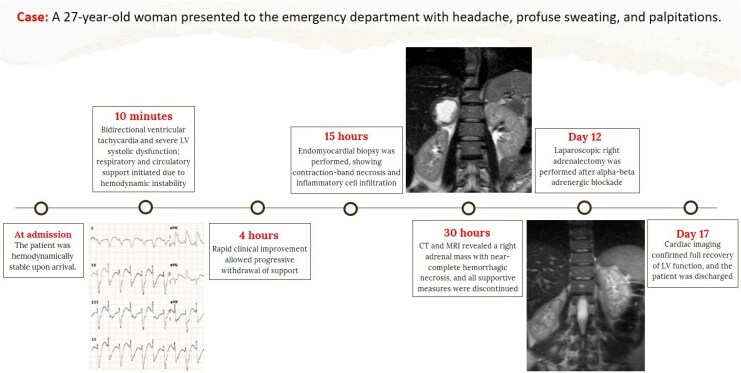


## Case report

A 27-year-old Caucasian woman was evaluated in the emergency department for headache, profuse sweating, and palpitations. She had a history of panic attacks as well as sporadic episodes of elevated blood pressure and heart rate. She had no history of chronic treatments or substance abuse.

Upon early evaluation, the patient was pale and diaphoretic, with a blood pressure of 140/100 mmHg and an oxygen saturation of 97%. Her physical examination revealed signs of pulmonary congestion and poor peripheral perfusion. Subsequently, she developed acute clinical deterioration requiring admission to the intensive care unit for haemodynamic instability secondary to cardiogenic shock. She was initiated on vasopressor support with norepinephrine and invasive mechanical ventilation due to respiratory failure.

**Figure 1:**
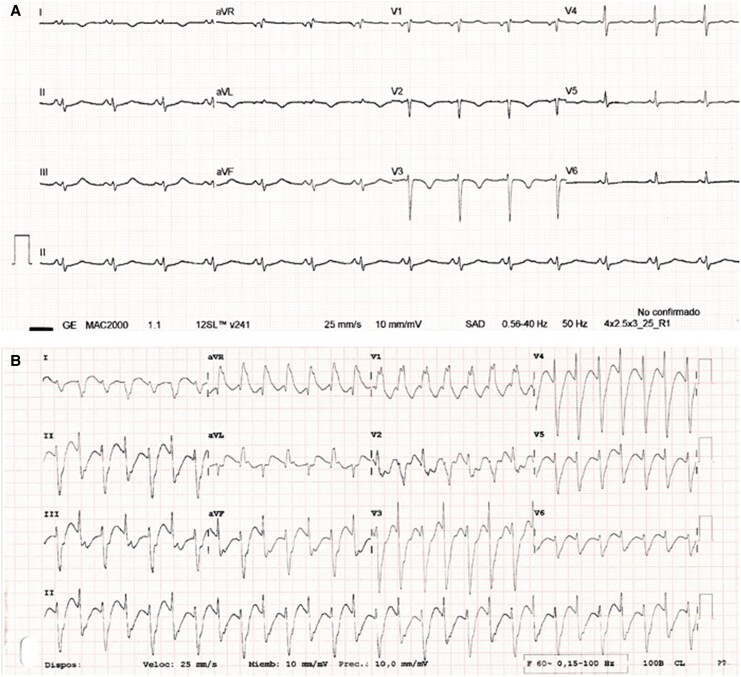
Twelve-lead electrocardiograms. Baseline in the emergency room (*A*). Ventricular tachycardia with beat-to-beat QRS axis shift, consistent with bidirectional ventricular tachycardia (*B*).

**Figure 2:**
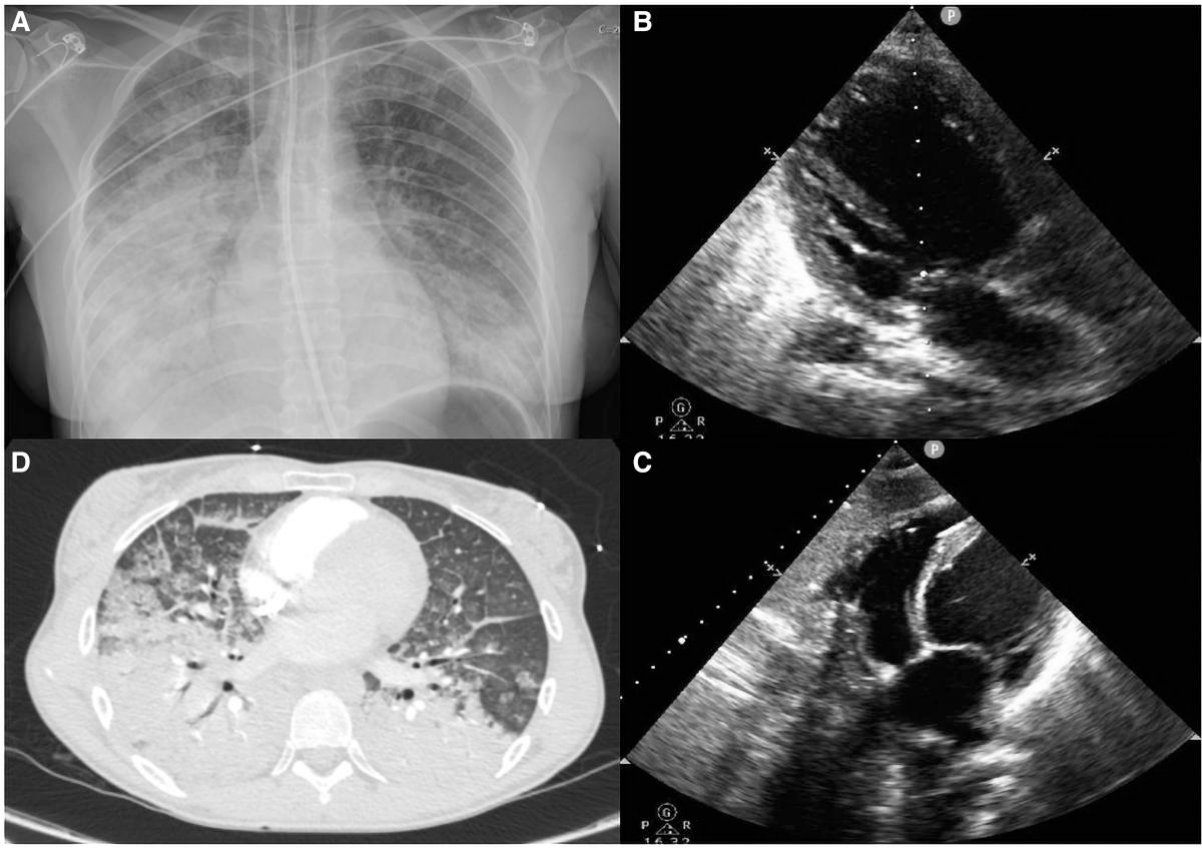
Bilateral pulmonary infiltrates, predominantly in the perihilar and basal regions, are visible on chest X-ray (*A*) and chest computed tomography (*D*), consistent with acute pulmonary oedema. Transthoracic echocardiography images showing a dilated left ventricle with severe systolic dysfunction (*B* and *C*).

Cardiac telemetry showed self-limiting episodes of ventricular tachycardia with beat-to-beat changes in the QRS complex axis, consistent with BVT, which was later confirmed by electrocardiogram (*Figure 1*). The chest X-ray showed perihilar and basal pulmonary infiltrates, consistent with acute pulmonary oedema (APE) (*Figure 2A*). The transthoracic echocardiogram revealed severe left ventricular dilatation with severe dysfunction and diffuse akinesia (*Figure 2B* and *C*).

Laboratory tests revealed leucocytosis with neutrophilia, hyperglycaemia, elevated D-dimer levels, high troponin T levels, metabolic acidosis, and elevated lactate levels. A computed tomography angiography ruled out pulmonary thromboembolism and confirmed the presence of APE (*Figure 2D*). The patient had no coronary stenosis on angiography, and the endomyocardial biopsy was negative for myocarditis but showed findings suggestive of catecholamine-induced cardiomyopathy (CICM), including contraction-band necrosis and inflammatory cell infiltration.

**Figure 3:**
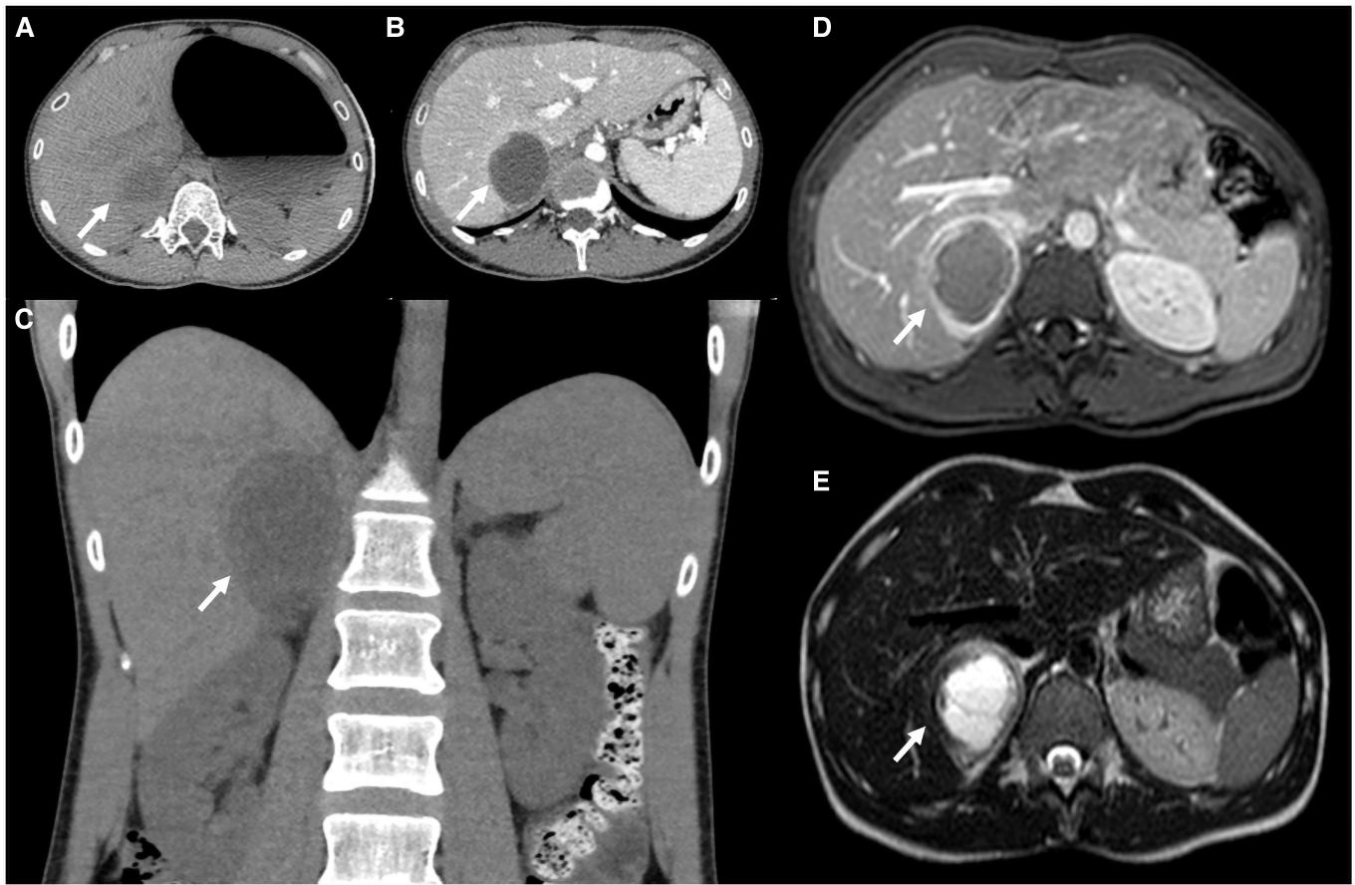
Abdominal computed tomography and magnetic resonance imaging. Axial computed tomography showing a large right adrenal mass measuring 7.1 × 6.6 cm (arrow) in sequences without contrast (*A*) and with contrast (*B*) with evidence of recent bleeding; coronal reconstruction (*C*) in computed tomography addressing the tumour (arrow); magnetic resonance imaging in T1 (*D*) and T2 (*E*) sequences showing the pheochromocytoma (arrow) with almost complete haemorrhagic necrosis.

Computed tomography revealed a heterogeneous mass measuring 7.1 × 6.6 cm in the right adrenal gland, with signs of recent haemorrhage (*Figure 3A–C*). These findings were confirmed by abdominal MRI, which was compatible with a pheochromocytoma showing haemorrhagic necrosis (*Figure 3D* and *E*). Of note, in the early hours after admission, the patient received high-dose vasopressors (norepinephrine) and inotropes (dobutamine), but subsequently showed rapid clinical improvement and was weaned from catecholamine support and invasive mechanical ventilation.

**Figure 4:**
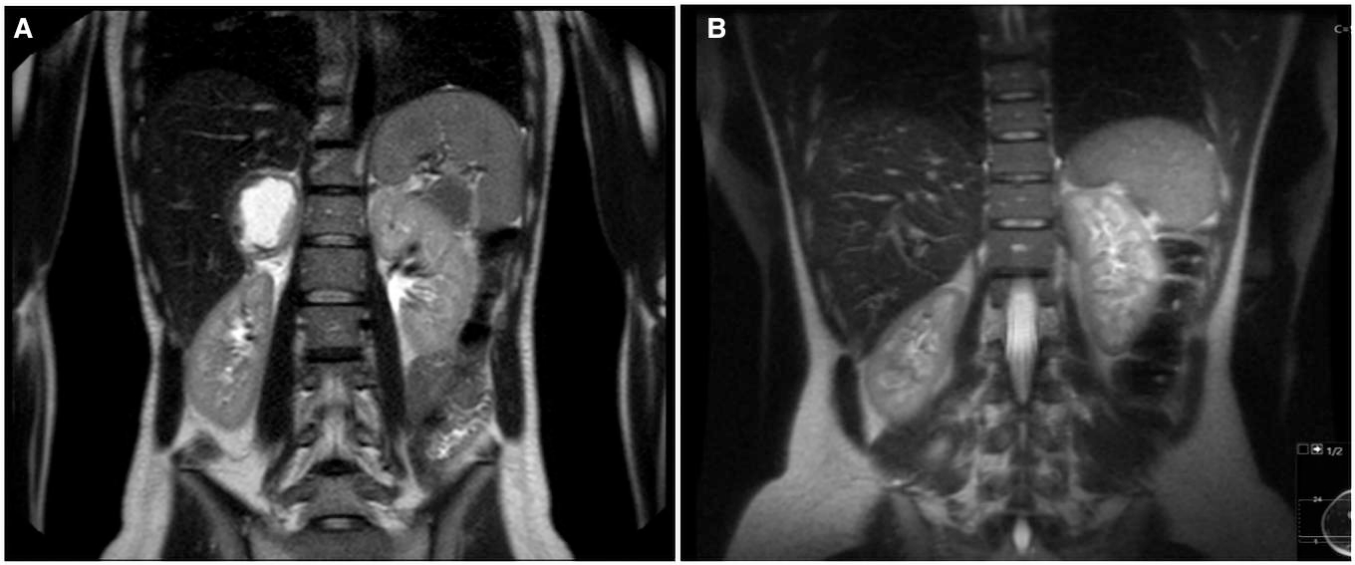
Pre-operative magnetic resonance imaging showing a right adrenal pheochromocytoma (arrow) (*A*) and follow-up imaging (*B*) showing absence of the tumour.

Due to the severity of the clinical presentation and the complementary tests, a right laparoscopic adrenalectomy was performed, after pre-operative preparation with alpha–beta adrenergic blockade (*Figure 4A* and *B*). The histopathological examination confirmed the diagnosis of pheochromocytoma.

The patient experienced a favourable outcome, with no recurrent arrhythmias and a complete recovery of the left ventricular ejection fraction. Genetic testing did not identify any associated mutations. She was later discharged without complications and has remained asymptomatic and normotensive during follow-up to date.

## Discussion

Bidirectional ventricular tachycardia is a form of PVT characterized by beat-to-beat alternation in the QRS axis.^4,8,9^ Although uncommon, it is life-threatening due to its tendency to compromise haemodynamic stability and degenerate into ventricular fibrillation.^4^

The underlying mechanisms of BVT are similar to those of other tachyarrhythmias, including increased automaticity, triggered activity, and reentry.^8^ Its generation and perpetuation depend on the specific aetiology, involving two morphologically distinct foci or circuits that alternate in a stable manner.^4,8^ It usually occurs in conditions associated with increased intracellular calcium, which predispose the appearance of late afterdepolarizations.^4,10^

Bidirectional ventricular tachycardia is most commonly associated with digitalis toxicity and CPVT.^2^ It has also been reported in ischaemic heart disease and other conditions characterized by elevated sympathetic tone.^8^ Because BVT is related to limited aetiologies, it is crucial to thoroughly investigate the underlying cause.^8^ In the case presented, the patient was not taking any medications, coronary angiography ruled out coronary stenosis, no electrolyte disturbances were identified, and her age was older than expected for CPVT, in addition to having no pertinent family history. This thorough investigation guided our treatment decisions and ultimately led to a successful outcome.

From a therapeutic point of view, the first step is to assess and recognize the unstable patient who will benefit from immediate intervention of the arrhythmia (such as defibrillation).^9^ Once stabilized, therapies targeting the primary cause are essential to ensure good outcomes.^8^

Pheochromocytoma is a rare tumour that produces catecholamines and arises from chromaffin cell in the adrenal medulla or paraganglia.^1^ It typically presents with the classic triad of headache, palpitations, and diaphoresis.^1^ In some cases, patients present with serious cardiovascular complications such as ventricular arrhythmias, ventricular dysfunction, or cardiogenic shock.^3,7,10^ Several mechanisms have been proposed to explain the pathophysiology of cardiovascular failure, including the direct myocytotoxic effect of increased catecholamine levels, higher myocardial oxygen demand due to tachycardia and elevated afterload, or coronary vasospasm caused by alpha-1 stimulation.^11,12^ Diagnosis is based on a combination of clinical and laboratory data, supplemented by imaging evidence of the tumour.^1^

Haemorrhagic necrosis of a pheochromocytoma rarely presents acutely but is often devastating.^13^ In our case, the massive release of catecholamines due to the haemorrhage resulted in cardiogenic shock. Although pheochromocytoma typically induces hypertension, 20% of patients may present with hypotension and 2% with cardiogenic shock.^5,7^ Its management is challenging because the therapies of choice (alpha–beta blockers) might be harmful in patients with haemodynamic instability. In addition, the treatment of cardiogenic shock (vasopressors and inotropes) may be less effective or even exacerbate cardiac dysfunction.^14^ Since myocardial stunning caused by excess catecholamines is mostly reversible, the implementation of mechanical circulatory support devices has proven effective in cases refractory to medical treatment.^15^

In this case, due to marked hypotension with tissue hypoperfusion, the patient required vasopressors and inotropics, as well as deep sedation, improving progressively without the need for mechanical support. With haemodynamic stability achieved, sequential alpha–beta blockade was initiated to control hypertension and regulate heart rate. Finally, a laparoscopic adrenalectomy was performed 10 days after the start of treatment to ensure the safety of the surgical procedure.

Although catecholamine excess is involved in both Takotsubo syndrome (TTS) and pheochromocytoma-related CICM, several differences exist. Takotsubo syndrome is usually triggered by acute emotional or physical stress, causing transient apical ballooning, whereas CICM results from sustained supraphysiological catecholamine exposure and may cause more extensive or biventricular damage. Endomyocardial biopsy findings can overlap (contraction-band necrosis, oedema, focal inflammation), but CICM may produce more persistent injury if untreated. Clinically, TTS predominantly affects postmenopausal women with generally favourable prognosis, whereas CICM can present severely and requires timely medical and surgical management.^16^

This clinical case highlights the diagnostic challenge posed by an atypical presentation of pheochromocytoma, with the presence of a BVT being a crucial finding that led to the diagnosis. Early recognition is essential, as patients presenting with cardiogenic shock have a poor prognosis without early aggressive and appropriate medical therapy.^6,7^

## Lead author biography


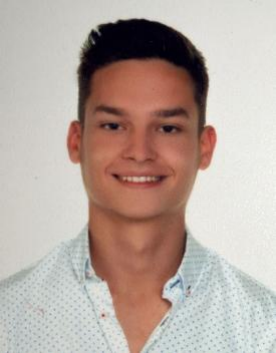


Alejandro Manuel López-Pena studied medicine at the University of Santiago de Compostela in Spain. He began his residency in cardiology in 2022. He is currently physician in residency in the Department of Cardiology at the Hospital Universitario Lucus Augusti in Lugo, Spain.

## Data Availability

The data underlying this article are available in the article.
